# The association of physical activity and sedentary behaviour on health-related quality of life: a cross-sectional study from the physical activity at work (PAW) trial

**DOI:** 10.1186/s44167-023-00031-7

**Published:** 2023-10-11

**Authors:** Katika Akksilp, Falk Müller-Riemenschneider, Yot Teerawattananon, Cynthia Chen

**Affiliations:** 1 Saw Swee Hock School of Public Health, National University of Singapore and National University Health System, Singapore, 117549 Singapore; 2 Health Intervention and Technology Assessment Programme (HITAP), Ministry of Public Health, Nonthaburi, Thailand; 3 Yong Loo Lin School of Medicine, National University of Singapore and National University Health System, Singapore, Singapore

**Keywords:** Sedentary behaviour, Physical activity, Health-related quality of life, EQ-5D

## Abstract

**Introduction:**

Physical inactivity and sedentary behaviour independently increase morbidity and negatively affect quality of life. This study evaluates the associations between physical activity and sedentary behaviour with health-related quality of life, including the five dimensions of quality of life (mobility, self-care, usual activities, pain or discomfort, and anxiety or depression).

**Methods:**

This cross-sectional study analysed baseline data from Thailand's Physical Activity at Work (PAW) trial. Physical activity data were collected using the ActiGraph™ accelerometer, worn on the right hip for a minimum of three ten-hour workdays. Accelerometer data were then used to categorise participants into: (i) not-sedentary and physically active (the Reference Group), (ii) not-sedentary but inactive, (iii) sedentary but active, and (iv) sedentary and inactive. We employed the EuroQol-5 dimensions questionnaire with five scoring levels (EQ-5D-5L) to measure health-related quality of life. The Thai EQ-5D-5D valuation was utilised to convert the EQ-5D profile into utility index scores (EQ-5D values). Tobit regression models were used to analyse EQ-5D value differences. Moreover, the odds of having problems in each EQ-5D dimension were compared between categories.

**Results:**

277 valid participant data were included. Older age (P = 0.007), higher education (P < 0.001), and higher prevalence of cardiovascular disease (P = 0.032) were observed in participants who were sedentary and physically inactive compared to other groups. We found − 0.0503 (95% CI: − 0.0946–− 0.00597) lower EQ-5D value and 1.39 (95% CI: 1.07–1.79) higher odds of reporting pain or discomfort problems in the sedentary and physically inactive group compared to the Reference Group. We also found 2.12 (95%CI: 1.14–5.40) higher odds of reporting usual activity problems in the not-sedentary but physically inactive group than in the Reference Group.

**Discussion:**

We found further evidence of the potential benefit of higher physical activity levels and lower sedentary time for higher quality of life among healthy office workers in Thailand. Further research with larger cohorts and longitudinal data is needed to establish a stronger foundation for interventions and economic evaluations targeting physical activity promotion to improve quality of life.

**Supplementary Information:**

The online version contains supplementary material available at 10.1186/s44167-023-00031-7.

## Introduction

Spending more time being sedentary, such as prolonged sitting or lying down, and less physical activity is associated with a greater risk of non-communicable diseases and all-cause mortality [1–3]. The World Health Organisation recommends that adults spend at least 150–300 min in moderate-intensity physical activity or 75–150 min in vigorous-intensity physical activity, or equivalence, per week and spend less time sedentary [4]. Many studies reported a negative association between health outcomes and sedentary behaviour [5]. Spending more time in physical activity provides a moderate protective effect against depression and a small protective effect against anxiety [6]. A recent review of Cochrane systematic reviews of randomised trials also concluded that exercise reduces mortality rates and improves quality of life among various populations, including children and adolescents, adults with and without underlying conditions such as heart diseases and mental illnesses, and the ageing population [7].

Moreover, recent studies in sedentary behaviour research introduce different constructs between physical activity and sedentary behaviour, indicating how “*sedentary behaviour may be more than just physical inactivity”* [8]. For instance, individuals devoting 30 min daily to exercise, thus meeting the criteria for being physically active, can still be deemed highly sedentary due to prolonged periods spent seated in front of monitors throughout the remainder of the day [9]. Evidence also shows independent, negative effects of sedentary behaviour on health [10, 11], increasing the risks of various diseases such as cardiovascular disease and type 2 diabetes [12, 13].

Health-related quality of life (HRQoL) reflects an individual’s physical, mental, and social well-being and can be measured using several tools [14]. One of the most widely used measures is the EuroQol-5 Dimension (EQ-5D, five-level version) [15]. The significant importance of incorporating EQ-5D HRQoL data collection and analysis in research is the use in cost-utility analysis for health technology assessment, which can provide context-specific evidence for policy evaluation and sustainability of implementation [16]. Globally, studies have reported positive associations between higher physical activity levels and HRQoL [17]. However, very few studies included the sedentary behaviour domain to explore the correlations on HRQoL [18].

While overall EQ-5D HRQoL provides the foundation for health technology assessment and priority setting in public health investment, a better understanding of EQ-5D dimensions can give valuable comprehension of HRQoL problems within the targeted population [16]. However, few studies have explored the effects of both physical activity and sedentary behaviour on each HRQoL dimension. A recent study found positive associations between self-report active and not-sedentary lifestyles on all HRQoL domains in adults during a COVID-19 outbreak [19]. Other studies focusing on older adults also reported parallel results where higher physical activity levels and lower sedentary time are correlated with better HRQoL in all dimensions [20–22]. Moreover, EQ-5D dimensions affect the overall HRQoL differently across countries. For example, the mobility dimension showed the greatest impact on utility decrement in the Thai, Korean, Japanese, Indonesian, and Canadian populations. In contrast, pain or discomfort and anxiety or depression were most significant for the Dutch and English populations [23].

In Thailand, more than 70% of all deaths are attributed to non-communicable diseases [24]. Physical inactivity alone contributes to 2.4% of all deaths in the country [25], where around 31% of Thai adults do not meet the recommended physical activity level [26]. Moreover, Thais spend a significant portion of their day, approximately 14 h, being sedentary [26]. In the Thai ageing population, a nationwide survey found that no regular exercise had the highest odds ratio of poor quality of life compared to hearing or sleeping difficulty or poor financial status [27]. Other studies reported that leisure, household, and work-related activities were associated with higher HRQoL [28, 29]. In addition, a cross-sectional survey found that performing, at least, three weekly exercise sessions improves HRQoL in Thai adults [30].

Nevertheless, there has not been any study in Thailand that evaluates the association between sedentary behaviour on overall or domains related to HRQoL. This is despite the increasing studies on physical activity and sedentary behaviour in recent years [31]. Furthermore, a recent scoping review of physical activity and sedentary behaviour research in Thailand encouraged researchers to use accelerometer data for more robust evidence since 94% of the studies used self-report data [31]. Thus, we used baseline data from the PAW study, a cluster-randomised control trial including 282 office workers in Thailand, which incorporated accelerometer-data measurement of physical activity and sedentary behaviour and self-report HRQoL using the EQ-5D-5L questionnaire [32]. This cross-sectional analysis evaluates the associations between physical activity and sedentary behaviour on HRQoL, including the five dimensions of HRQoL.

## Methods

This is a sub-study of the PAW cluster-randomised trial with multi-component intervention, including individual (pedometer and individual-based weekly lottery reward), social (team movement breaks and team-based weekly lottery reward), organisational (leaders’ involvements), and environmental level (posters) to reduce sedentary time and increase physical activity in Thai office workers. Detailed protocol [32] and the main results of the trial [33] are available online. Eighteen offices in the Ministry of Public Health, Thailand, were recruited between July to September 2020. The recruitment criteria included: i) aged at least 18 years old, ii) were not pregnant, iii) had no physical limitation to perform team movement breaks. The baseline data were collected by the PAW-study research team, which consisted of trained research staffs and programme managers, at participants’ office buildings between July and October 2020 before participants were randomised into the 6-month active intervention and control group.

## Measures

### Physical activity and sedentary behaviour

Participants were requested to wear the ActiGraph™ wGT3X-BT triaxial accelerometer (ActiGraph, Pensacola, Florida, USA) on the right waist as much as possible (except for bathing, swimming, or diving) for ten days. A validity wear time criterion of more than 10 h per day for at least three workdays was used. Participants with insufficient wear time were asked to re-wear the accelerometers [32]. We used the ActiLife software (Version 6.13.4) to extract count data from the accelerometer and then R package ‘PhysicalActivity’ to categorise the tri-axial accelerometer data into time spent in sedentary behaviour (150 and below counts per minute), light physical activity (151 to 2689 counts per minute), moderate physical activity (2690 to 6167 counts per minute) and vigorous physical activity (6168 and above counts per minute), according to Freedson cutpoints and a validation study [34–36]. The daily mean time spent in sedentary behaviour and moderate-to-vigorous physical activity were then calculated. Next, participants were grouped into ‘sedentary’ or ‘not-sedentary’, using a cut-off of nine hours per day or more spent in sedentary behaviour [37]. ‘Physically active’ was defined as having at least 150 min spent in moderate physical activity or 75 min spent in vigorous physical activity per week [4]. Finally, we categorised participants into four categories: (i) not-sedentary and physically active (the Reference Group), (ii) not-sedentary but physically inactive, (iii) sedentary but physically active, and (iv) sedentary and physically inactive.

### Health-related quality of life

This study measured HRQoL using the EuroQol-5 Dimension questionnaire with five scoring options (EQ-5D-5L). The five dimensions included mobility, self-care, usual activities, pain or discomfort, and anxiety or depression [38]. Different health profiles from the questionnaire were summarised into utility index scores (EQ-5D values) using the Thai valuation study [23]. Moreover, to analyse between-group differences in each dimension, the five scoring options (no problem, mild-, moderate-, severe problem, and unable to perform tasks) were categorised into either “having no problems” (including only if the answer was ‘no problem’) or “having problems” (including ‘mild-, moderate-, severe problems, and unable to perform tasks) [16, 20].

### Covariates

Age, sex, education, smoking history, and underlying cardiovascular disease data were collected using the Thai National Statistical Office's health survey [32, 39]. Although the survey has been commonly utilised in previous studies [40–43], no validation study was conducted. Nevertheless, we employed the questionnaire because it has been extensively used in health-related research in Thailand, allowing us to compare our results with findings from other studies effectively. The total duration of each interview was approximately 30 min per participant.

Education, smoking history, and underlying cardiovascular diseases data were categorised as binary data, with education classified as above Bachelor's degree or below, smoking history categorised as ever or never smoked, and underlying conditions categorised as having any cardiovascular-related disease (including diabetes mellitus, hypertension, dyslipidemia, or any heart disease) or having none. A physical examination was done to collect body-mass index data. We measured height to the nearest 0.1 cm and weight to the nearest 0.1 kg to calculate participants' body-mass index (weight (kg.)/height (m.)^2^). Participants were classified as obese and not obese, using the Asian body-mass index cut point of 25 kg/m^2^ [44].

### Statistical analysis

Participant characteristics were summarised and compared between participants’ physical activity and sedentary behaviour categories using mean (SD) with t-test for continuous variables, and count (percentage) with Pearson’s chi-squared test for categorical variables. In addition, descriptive analyses, such as mean (SD), median of EQ-5D values, the proportion of participants without problems, and data visualisation, were also implemented to comprehensively understand participants’ cross-sectional overall HRQoL and quality of life by dimensions [16].

The primary analysis used Tobit regression models to account for ceiling values [45] and examine differences in EQ-5D values between each participant category compared to the Reference Group. Four different models were performed: (i) the unadjusted model, (ii) adjusted for sex, age, smoking, and obesity, (iii) adjusted further for education, and (iv) finally adjusted further for underlying cardiovascular diseases. In addition, by stratifying the time spent in physical activity and sedentary behaviour into working and leisure hours, a supplementary analysis using Tobit regression models with continuous exposure variables was conducted to explore their associations on EQ-5D values.

The odds of reporting “having problems” in each EQ-5D dimension were estimated by comparing each participant’s physical activity and sedentary behaviour categories to the Reference Group, using the unadjusted and the fully adjusted logistic regression models (adjusted for sex, age, smoking, obesity, and underlying cardiovascular diseases).

Sensitivity analyses were performed using distinct binary variables for sedentary behaviour (sedentary vs not-sedentary participants, without the physical activity component) and physical activity (physically inactive vs active participants without the sedentary behaviour component) as exposure variables in both Tobit and logistic regression analyses. All statistical data analyses were performed using Stata software version 14.2, with a significance level of 5%.

## Results

### Participant characteristics

Of 282 PAW participants, 277 (98.2%) valid accelerometer-measured data were collected. Participant characteristics are presented in Table 1 across the four categories: (i) the Reference Group (*n* = 97), (ii) not-sedentary but physically inactive (*n* = 91), (iii) sedentary but physically active (*n* = 36), and (iv) sedentary and physically inactive (*n* = 53). Demographic data were not balanced among categories, especially for age (P = 0.007), sex (P < 0.001), education status (P < 0.001), and underlying cardiovascular diseases (P = 0.032). Older age, higher education, and higher prevalence of cardiovascular disease were observed in participants who were sedentary and physically inactive (Table 1).

**Table 1:** Participant characteristics, physical activity levels, and EQ-5D values

	Total	Non-Sedentary^a^ and Active^b^	Non-Sedentary but Inactive^b^	Sedentary^a^ but Active	Sedentary and Inactive	P-value
	n = 277	n = 97	n = 91	n = 36	n = 53	
Age, year	38.7 (10.3)	37.4 (8.91)	38.1 (10.5)	37.1 (9.25)	43.0 (11.9)	0.007
Gender, female	225 (81.2%)	69 (71.1%)	85 (93.4%)	24 (66.7%)	47 (88.7%)	< 0.001
Education, above Bachelor’s degree	98 (35.4%)	36 (37.1%)	20 (22.0%)	12 (33.3%)	30 (56.6%)	< 0.001
Smoking	16 (5.78%)	7(7.22%)	6 (6.59%)	2 (5.56%)	1 (1.89%)	0.581
Obese (Body mass index ≥ 25 kg/m^2^)	64 (23.1%)	24 (24.7%)	17 (18.7%)	8 (22.2%)	15 (28.3%)	0.579
Having any cardiovascular disease	38 (13.7%)	8 (8.25%)	14 (15.4%)	3 (8.33%)	13 (24.5%)	0.032
**Physical activity**						
Moderate-to-Vigorous physical activity per week, min	175 (126)	270 (116)	89.5 (35.5)	269 (132)	84.8 (31.2)	< 0.001
Sedentary time per day, min	486 (109)	441 (80.7)	422 (83.8)	596 (51.8)	605 (52.5)	< 0.001
**EuroQol-5 dimension values**						
Mean (SD)	0.910 (0.102)	0.923 (0.111)	0.907 (0.0893)	0.921 (0.0670)	0.887 (0.122)	0.001
Median	0.928	0.960	0.928	0.928	0.924	
**EuroQol-5 dimensions: participants with problems in**^c^						
Mobility	99 (35.7%)	29 (29.9%)	33 (36.3%)	14 (38.9%)	23 (43.4%)	0.398
Self-care	18 (6.50%)	4 (4.12%)	6 (6.59%)	1 (2.78%)	7 (13.2%)	0.130
Usual Activity	59 (21.3%)	14 (14.4%)	24 (26.4%)	6 (16.7%)	15 (28.3%)	0.105
Pain or Discomfort	154 (55.6%)	48 (49.5%)	49 (53.9%)	20 (55.6%)	37 (69.8%)	0.116
Anxiety or Depression	128 (46.2%)	44 (45.4%)	42 (46.2%)	17 (47.2%)	25 (47.2%)	0.996

Categorical variables are expressed in count (percentage); Continuous variables are expressed in mean (standard deviation)

^a^Sedentary refers to spending at least nine hours per day in sedentary behaviours, while not-sedentary refers to spending less than nine hours per day in sedentary activities

^b^Physically inactive refers to participants who did not meet the current physical activity guideline (≥ 150 min moderate-intensity or > 75 min vigorous-intensity equivalent physical activity per week), while active refers to participants who met the guideline

^c^Participants were categorised into either ‘Having problem’ (1) or ‘Having no problem’ (0) in each dimension

**Fig. 1:**
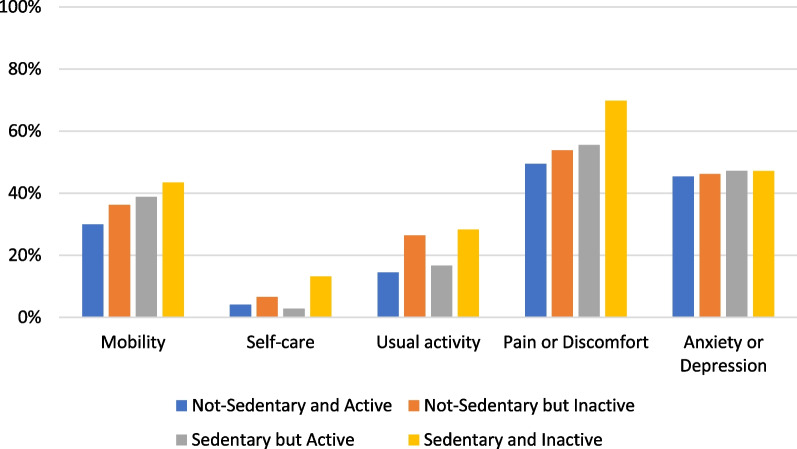
Health-related quality of life dimensions^a^ compared between physical activity and sedentary behaviour categories. ^a^Health-related quality of life dimensions was collected using EQ-5D-5L interviewer-administered questionnaire. Participants were categorised into either ‘Having problem’ (1) or ‘Having no problem’ (0) in each dimension. ^b^Sedentary refers to spending at least nine hours per day in sedentary behaviours, while not-sedentary refers to spending less than nine hours per day in sedentary activities. ^c^physically inactive refers to participants who did not meet the current physical activity guideline (≥150 minutes moderate-intensity or >75 minutes vigorous-intensity equivalent physical activity per week), while active refers to participants who met the guideline

Overall, participants had high EQ-5D values (mean = 0.910, SD = 0.102). Participants who were sedentary and physically inactive had the lowest mean EQ-5D values of 0.887 (SD = 0.122). In contrast, participants in the Reference Group had the highest mean EQ-5D values of 0.923 (SD = 0.111). Significant mean differences in EQ-5D values were observed among participant categories (P = 0.001). Participants who were sedentary and physically inactive were more likely to have problems with mobility (43.4% vs 29.9%), usual activity (28.3% vs 14.4%), and pain or discomfort (69.5% vs 49.5%) compared to the Reference Group, though not statistically significant (Table 1, Fig. 1).

### Differences in EQ-5D values

From the Tobit regression analyses, all categories of participants had lower EQ-5D values than the Reference Group. Sedentary and physically inactive participants had -0.0503 (95%CI: − 0.0946 to − 0.00597) lower EQ-5D values than the Reference Group. All other findings from the Tobit regression analyses were non-significant.

Sensitivity analyses showed consistent findings. When comparing distinctly physically inactive (n = 144) to active participants (n = 133), excluding the sedentary data component, inactive participants had − 0.0326 (95%CI: − 0.0634 to − 0.00187) lower EQ-5D values in the unadjusted model (Additional file 1: Table S1). Similarly, a lower EQ-5D value was observed in the sedentary (n = 89) compared to the not-sedentary participants (n = 188), although the differences did not reach statistical significance (Additional file 1: Table S2).

Parallel results were also observed when stratifying physical activity and sedentary behaviour levels in working and leisure hours and using them as continuous variables. Spending one more hour in moderate-to-vigorous physical activity was associated with 0.0520 (95%CI: 0.000792 – 0.103) increase in EQ-5D value in waking hours in the unadjusted model. A lower EQ-5D value was associated with higher sedentary time, whereas a higher EQ-5D value was associated with higher physical activity levels in both working and leisure hours, although without statistical significance. Nevertheless, we observed a higher magnitude of associations during leisure hours than during working hours for sedentary time and time spent in light physical activity on EQ-5D value (Additional file 1: Table S3).

### Differences in EQ-5D dimensions

Considering all the five dimensions of the EQ-5D-5L, participants who were not-sedentary but physically inactive, sedentary but active, and sedentary and inactive had higher odds of reporting problems in each EQ-5D dimension than the Reference Group (Table 3). The sedentary and physically inactive participants had the highest odds ratio of having pain or discomfort problems (OR 1.39 compared to the Reference Group, with 95%CI: 1.07 to 1.79, from the adjusted model) (Table 3). Moreover, not-sedentary but physically inactive participants had 2.49 (95%CI: 1.14 to 5.40) higher odds of having problems conducting usual activity than the Reference Group.

Compared distinctly between physically inactive and active participants without the sedentary data component in the sensitivity analysis, more inactive participants reported having problems than the active participants in all dimensions (Additional file 1: Fig. S1). Without statistical significance, the physically inactive group observed 1.89 (95%CI: 0.982 to 3.62) higher odds of having usual activity problems (Additional file 1: Table S4). Accordingly, sedentary participants had higher odds ratios compared to not-sedentary participants (Additional file 1: Fig. S2), with 1.72 (95%CI: 1.01 to 2.94) higher odds of having pain or discomfort problems than not-sedentary participants (Additional file 1: Table S5).

## Discussion

This cross-sectional study used accelerometer-measured physical activity and sedentary behaviour data from the PAW cluster-randomised trial [33] to evaluate their associations with EQ-5D-5L HRQoL. The results showed that participants who were sedentary and physically inactive had lower EQ-5D values than those who were not-sedentary and active. Moreover, higher odds of reporting problems in the usual activity and pain or discomfort dimensions were found in participants who were either sedentary or physically inactive compared to not-sedentary and active.

The findings parallel previous studies that reported a positive impact of physical activity on HRQoL in adult populations in different countries [46, 47]. A recent study reported a significant association between insufficient physical activity and lower physical HRQoL. However, the study did not find significant associations between physical activity on the mental dimension of HRQoL, or sedentary behaviour on any HRQoL domain [5]. Another recent review of systematic reviews indicated that physical activity improves HRQoL and well-being, with the most robust evidence in older adults and strong evidence in the adult population [17]. Regarding the negative impact of sedentary behaviour on HRQoL, a systematic review reported that higher levels of sedentary behaviours are related to lower physical HRQoL, while unclear evidence was found in mental and social HRQoL domains [18]. From our analyses, all categories of participants (sedentary and physically inactive, sedentary but active, and non-sedentary but inactive) had lower EQ-5D values than not-sedentary and active, based on the current recommendations from physical activity guidelines [4, 10]. The lowest EQ-5D value was the participants who were sedentary and inactive. The results indicated that a higher HRQoL is related to both higher physical activity and lower sedentary behaviour levels in adult office workers. Our results align with another recent study concludingthat an extended period of sedentary time could diminish the mitigating impact of moderate-to-vigorous physical activity on the vulnerability to the risk of poor HRQoL [48]. The rationale behind the correlations found in our study might be based on the negative impact on health of sedentary behaviour and the lack of physical activity, attributing to various non-communicable diseases [1–3, 12] and also psychiatric conditions [6, 49, 50]. On the other hand, this might be due to the reverse causation, where experiences of pain or discomfort impede individuals from exercise and enhance sedentary behaviours.

Furthermore, the positive associations of higher physical activity levels on HRQoL were observed in both working and leisure hours (Additional file 1: Table S3). This contrasts with the concept of the physical activity paradox, where emerging evidence reports a negative impact of occupational physical activity on health [51–53]. A plausible rationale for this observation could be that our participants were office workers who did not engage in strenuous physical tasks. On the other hand, studies supporting the physical activity paradox defined higher-risk categories as ‘heavy physical work (or labour)’, ‘carrying heavy burdens…’, and ‘activities that could significantly elevate heart rate during working hours’ [51, 52, 54]. Hence, our participants, categorised as sedentary workers, may not face the same detrimental effects from increased physical activity during their working hours.

To date, only a limited number of studies have analysed the associations between sedentary and physical activity levels on different HRQoL dimensions. Such analysis provides insightful information and shows how the focused explanatory variables associate differently with each HRQoL dimension in different populations [16]. Previous studies in ageing populations reported moderate-to-strong associations between increased physical activity levels and improved HRQoL across all dimensions [21, 22, 55]. Studies which included both physical activity and sedentary levels reported comparable results. In the U.S. adults, a study reported significant correlations between the poor physical health and activity limitation domains with both lower moderate-to-vigorous physical activity and higher sedentary behaviour levels, while all domains correlated with moderate-to-vigorous physical activity level alone [48]. On the other hand, another study in the ageing Korean population found significant associations across all dimensions, with the highest odds ratio in problems performing usual activities [20]. These pieces of evidence aligned with our study among healthy adult office workers, where higher sedentary and lower physical activity levels were associated with higher odds of having problems in HRQoL dimensions, particularly in the pain or discomfort dimension (Table 3). The rationale behind our findings might be due to the connection between high sitting time and pain, as reported in previous studies [56, 57]. Another possible explanation could be attributed to the highest occurrence of pain or discomfort problems, as compared to other domains, in our population of healthy office workers. This contributed to a greater power to detect statistical significance among participant categories (Table 2–3).

**Table 2:** Differences in EQ-5D values between physical activity and sedentary behaviour categories

	Unadjusted model	Adjusted model A	Adjusted model B	Adjusted model C
	Beta (95%CI)	Beta (95%CI)	Beta (95%CI)	Beta (95%CI)
Not-sedentary^a^ and Active^b^ (n = 97)	Reference	Reference	Reference	Reference
Non-Sedentary but Inactive^b^	− 0.0241	− 0.0223	− 0.0238	− 0.0218
(n = 91)	(− 0.0615–0.0133)	(− 0.0602–0.0156)	(− 0.0621–0.0145)	(− 0.0603–0.0167)
Sedentary^a^ but Active	− 0.0108	− 0.0132	− 0.0136	− 0.0133
(n = 36)	(− 0.0607–0.0390)	(− 0.0623–0.0358)	(− 0.0626–0.0355)	(− 0.0623–0.0356)
Sedentary and Inactive	− 0.0547**	− 0.0541**	− 0.0529**	− 0.0503**
(n = 53)	(− 0.0979–− 0.0114)	(− 0.0979–− 0.0103)	(− 0.0969–− 0.0089)	(− 0.0946–− 0.00597)
Female		− 0.0200	− 0.0196	− 0.0208
		(− 0.00625–0.0226)	(− 0.0633–0.0230)	(− 0.0634–0.0218)
Age (year)		0.0003	0.0003	0.0006
		(− 0.00126–0.00182)	(− 0.00123–0.00187)	(− 0.00109–0.00220)
Obese (BMI ≥ 25 kg/m^2^)		− 0.0384**	− 0.0388**	− 0.0343*
		(− 0.0748–− 0.00195)	(− 0.0753–− 0.0024)	(− 0.0722–0.0035)
Smoking		− 0.0635*	− 0.0660*	− 0.0672*
		(− 0.131–0.00345)	(− 0.134–0.00166)	(− 0.135–0.0004)
Highest education: above bachelor’s degree			− 0.0084	− 0.0094
			(− 0.0417–0.0248)	(− 0.0426–0.0239)
Having any cardiovascular disease				− 0.0211
				(− 0.0709–0.0287)
Observations	277	277	277	277

Adjusted model A; adjusted for sex and age

Adjusted model B; adjusted for sex, age, obesity, and smoking history

Adjusted model C; adjusted for sex, age, obesity, smoking history, education, and cardiovascular diseases

^a^Sedentary refers to spending at least nine hours per day in sedentary behaviours, while not-sedentary refers to spending less than nine hours per day in sedentary activities

^b^Physically inactive refers to participants who did not meet the current physical activity guideline (≥ 150 min moderate-intensity or > 75 min vigorous-intensity equivalent physical activity per week), while active refers to participants who met the guideline

*p < 0.10, **p < 0.05

**Table 3:** Odds of having problems in each of the EeuroQol-5 dimensions^a^ among categories

	Mobility	Self-care	Usual activity	Pain or Discomfort	Anxiety or Depression
	OR (95%CI)	OR (95%CI)	OR (95%CI)	OR (95%CI)	OR (95%CI)
Unadjusted models					
Not-sedentary^b^ and Active^c^ (n = 97)	Reference	Reference	Reference	Reference	Reference
Not-sedentary but Inactive (n = 91)	1.33	1.64	2.12**	1.19	1.03
	(0.725–2.45)	(0.448–6.02)	(1.02–4.42)	(0.672–2.11)	(0.581–1.83)
Sedentary but Active (n = 36)	1.22	0.815	1.09	1.13	1.04
	(0.819–1.82)	(0.268–2.48)	(0.646–1.83)	(0.769–1.66)	(0.708 –1.52)
Sedentary and Inactive (n = 53)	1.22*	1.52*	1.33**	1.33**	1.02
	(0.964–1.53)	(0.995–2.33)	(1.01–1.75)	(1.05–1.69)	(0.819–1.28)
Adjusted models^d^					
Not-sedentary and Active (n = 97)	Reference	Reference	Reference	Reference	Reference
Not-sedentary but Inactive (n = 91)	1.21	1.21	2.36**	1.28	1.22
	(0.612–2.41)	(0.275–5.30)	(1.04–5.34)	(0.685–2.38)	(0.651–2.27)
Sedentary but Active (n = 36)	1.25	0.827	1.09	1.14	1.06
	(0.831–1.89)	(0.263–2.60)	(0.630–1.88)	(0.771–1.68)	(0.716–1.58)
Sedentary and Inactive (n = 53)	1.11	1.29	1.22	1.38**	1.01
	(0.852–1.44)	(0.785–2.12)	(0.890–1.67)	(1.06–1.79)	(0.787–1.29)

^a^Participants were categorised into either ‘Having problem’ (1) or ‘Having no problem’ (0) in each dimension

^b^Sedentary refers to spending at least nine hours per day in sedentary behaviours, while not-sedentary refers to spending less than nine hours per day in sedentary activities

^c^physically inactive refers to participants who did not meet the current physical activity guideline (≥ 150 min moderate-intensity or > 75 min vigorous-intensity equivalent physical activity per week), while active refers to participants who met the guideline

^d^Estimates were adjusted for sex, age, obesity, smoking history, education, and cardiovascular diseases

*p < 0.10, **p < 0.05

## Strengths and limitations

Major strengths of the study were the higher accuracy of the physical activity and sedentary data due to standard tri-axial accelerometer-measure data collection [35, 58], the specific Thai value set for analysing the EQ-5D values tailored for the Thai population [23], and the Tobit regression model to account for the ceiling effect of HRQoL in our healthy population [45]. Moreover, delving into different HRQoL domains generates ideas on how, in Thai office workers, being physically inactive and sedentary might be associated more with pain or discomfort, and less with other domains, such as anxiety or depression (Table 3). Nevertheless, there are several limitations of the study. First, this was secondary data analysis. The PAW study was initially designed to evaluate the effectiveness of a complex intervention in reducing sedentary time in office workers [32]. As such, this analysis, focusing on HRQoL, was not powered to detect statistical differences, resulting in lower generalisability of the associations. The second limitation is the cross-sectional design, preventing the determination of causality. Future studies with longitudinal data to estimate the causation of physical activity and sedentary behaviour on HRQoL are needed. For instance, a recent study in the Korean population reported that HRQoL in the early ageing population was affected by the change in physical activity level over an 8-year follow-up [59]. Another limitation is the inability to judge the importance or imply concrete meanings of the between-group EQ-5D values difference without further information. This is because populations with different health states have different scales for EQ-5D values [16]. The idea of Minimal Importance Differences calculation to determine the smallest difference in EQ-5D value that the population perceive as important has been discussed without consensus [60]. Nevertheless, the implication of EQ-5D values lies in future cost-utility analyses of the same population [16]. The last limitation is that the generalisability might be low because different countries have different contexts and also use different EQ-5D valuations. Similar studies in other countries should be conducted to understand the generalisability of the findings.

## Conclusion

This study underscored the importance of promoting physical activity along with reducing sedentary behaviour to enhance Thai office workers’ quality of life across different domains. Further research, incorporating larger cohorts and longitudinal data, is essential to establish a stronger foundation for interventions and economic evaluations targeting sedentary reduction and physical activity promotion for quality of life improvement in Thailand and beyond.

## Supplementary Information

**Additional file 1. Table S1.** The difference in EQ-5D value between physically inactive compared to active participants. **Table S2.** The difference in EQ-5D value between sedentary compared to not sedentary participants. **Table S3.** Tobit regression analysis of EQ-5D values with different exposures. **Table S4. **Odds of having problems in each of the EeuroQol-5 Dimensions between physically inactive compared to active participants. **Table S5.** Odds of having problems in each of the EeuroQol-5 Dimensions between sedentary compared to not-sedentary participants. **Figure S1.** Participants reporting problems in each of the EeuroQol-5 Dimensions compared between physically inactive and active participants. **Figure S2.** Participants reporting problems in each of the EeuroQol-5 Dimensions compared between sedentary and not-sedentary participants.

## Data Availability

The research team will have exclusive rights to the de-identified data for 24 months after the trial is completed. After that, the data and full protocol will be publicly accessible on the HITAP website.

## Abbreviation

BMI: Body mass index
CM: Centimetre
EQ-5D: : EuroQol’s-5 dimension
HRQoL: : Health-related quality of life
PAW: : Physical activity at work
