# Supplementary material for: The association of physical activity and sedentary behaviour on health-related quality of life: a cross-sectional study from the physical activity at work (PAW) trial
Source: J Act Sedentary Sleep Behav. 2023 Oct 11;2:22. doi: 10.1186/s44167-023-00031-7 (PMC11960397; doi:10.1186/s44167-023-00031-7)
Supplement: Supplementary file 1 — Additional file 1. Table S1. The difference in EQ-5D value between physically inactive compared to active participants. Table S2. The difference in EQ-5D value between sedentary compared to not sedentary participants. Table S3. Tobit regression analysis of EQ-5D values with different exposures. Table S4. Odds of having problems in each of the EeuroQol-5 Dimensions between physically inactive compared to active participants. Table S5. Odds of having problems in each of the EeuroQol-5 Dimensions between sedentary compared to not-sedentary participants. Figure S1. Participants reporting problems in each of the EeuroQol-5 Dimensions compared between physically inactive and active participants. Figure S2. Participants reporting problems in each of the EeuroQol-5 Dimensions compared between sedentary and not-sedentary participants. [file 44167_2023_31_MOESM1_ESM.docx]

**Additional file 1**

| **Table S1.** The difference in EQ-5D value between physically inactive compared to active participants ^a^ | | | | |
| --- | --- | --- | --- | --- |
|  | Unadjusted model | Adjusted for +Demographics | Adjusted for  +Education | Adjusted for  + Disease |
|  | beta (SE) | beta (SE) | beta (SE) | beta (SE) |
|  |  |  |  |  |
| Active | reference | reference | reference | reference |
| (n = 133) |  |  |  |  |
| Inactive | **-0.0326**^**^ | -0.0307^*^ | -0.0313^*^ | -0.0290^*^ |
| (n = 144) | (-0.0634 –  -0.00187) | (-0.0624 –  0.000905) | (-0.0630 –  0.000351) | (-0.0610 –  0.00295) |
| Female |  | -0.0171 | -0.0168 | -0.0181 |
|  |  | (-0.0597 –  0.0254) | (-0.0593 –  0.0257) | (-0.0606 –  0.0244) |
| Age (year) |  | 0.0001 | 0.000190 | 0.000437 |
|  |  | (-0.00143 –  0.00162) | (-0.00135 –  0.00173) | (-0.00120 –  0.00207) |
| Obese (BMI ≥ 25 kg/m^2^) |  | **-0.0396**^**^ | **-0.0401**^**^ | -0.0354^*^ |
|  |  | (-0.0760 –  -0.00312) | (-0.0766 –  -0.00366) | (-0.0733 –  0.00247) |
| Smoking |  | -0.0596^*^ | -0.0639^*^ | -0.0651^*^ |
|  |  | (-0.127 –  0.00743) | (-0.132 –  0.00391) | (-0.133 –  0.00256) |
| Highest education: above bachelor’s degree |  |  | -0.0132 | -0.0141 |
|  |  |  | (-0.0456 –  0.0192) | (-0.0465 –  0.0183) |
| Having any cardiovascular disease |  |  |  | -0.0222 |
|  |  |  |  | (-0.0720 –  0.0276) |
| Observations | 277 | 277 | 277 | 277 |
| ^a^ physically inactive refers to participants who did not meet the current physical activity guideline (≥150 minutes moderate-intensity or >75 minutes vigorous-intensity equivalent physical activity per week), while active refers to participants who met the guideline  ^*^ *p* < 0.10, ^**^ *p* < 0.05 | | | | |

| **Table S2.** The difference in EQ-5D value between sedentary compared to not sedentary participants ^a^ | | | | |  |
| --- | --- | --- | --- | --- | --- |
|  | Unadjusted model | Adjusted for +Demographics | Adjusted for  +Education | Adjusted for  + Disease | |
|  | beta (95%CI) | beta (95%CI) | beta (95%CI) | beta (95%CI) | |
|  |  |  |  |  | |
| Not Sedentary | reference | reference | reference | reference | |
| (n = 188) |  |  |  |  | |
| Sedentary | -0.0255 | -0.0268 | -0.0256 | -0.0247 | |
| (n = 89) | (-0.0583 – 0.00729) | (-0.0595 – 0.00578) | (-0.0586 –  0.00734) | (-0.0576 –  0.00818) | |
| Female |  | -0.0301 | -0.0299 | -0.0305 | |
| (vs. Male) |  | (-0.0713 –  0.0111) | (-0.0712 –  0.0113) | (-0.0716 –  0.0105) | |
| Age (year) |  | 0.000132 | 0.000181 | 0.000497 | |
|  |  | (-0.00140 –  0.00167) | (-0.00137 – 0.00173) | (-0.00115 –  0.00214) | |
| Obese (BMI ≥ 25 kg/m^2^) |  | **-0.0392**^**^ | **-0.0396**^**^ | -0.0337^*^ | |
|  |  | (-0.0757 –  -0.000262) | (-0.0762 –  -0.00297) | (-0.0769 –  0.00424) | |
| Smoking |  | -0.0658^*^ | **-0.0682**^**^ | **-0.0695**^**^ | |
|  |  | (-0.133 –  0.00154) | (-0.136 –  -0.000175) | (-0.137 –  0.00164) | |
| Highest education: above bachelor’s degree |  |  | -0.00798 | -0.0093 | |
|  |  |  | (-0.0408 –  0.0249) | (-0.00421 –  0.0235) | |
| Having any cardiovascular disease |  |  |  | -0.0276 | |
|  |  |  |  | (-0.0769 –  0.0217) | |
| Observations | 277 | 277 | 277 | 277 | |
| ^a^ Sedentary refers to spending at least nine hours per day in sedentary behaviours, while not-sedentary refers to spending less than nine hours per day in sedentary activities  ^*^ *p* < 0.10, ^**^ *p* < 0.05 | | | | |  |

| **Table S3** Tobit regression analysis of EQ-5D values with different exposures | | |
| --- | --- | --- |
|  | Unadjusted model | Adjusted model |
| Time spent in different activities (hour/day) | beta (95%CI) | beta (95%CI) |
| **Waking hours** |  |  |
| Sedentary behaviour | -0.000442  (-0.00891 – 0.00803) | -0.000918  (-0.00952 – 0.00768) |
| Light physical activity | 0.00709  (-0.00367 – 0.0178) | 0.00725  (-0.00409 – 0.0186) |
| Moderate-to-vigorous physical activity | **0.0520****  (0.000792 – 0.103) | 0.0493*  (-0.00388 – 0.103) |
| **Working hours** |  |  |
| Sedentary behaviour | -0.0153  (-0.0347 – 0.00412) | -0.0138  (-0.0334 – 0.00580) |
| Light physical activity | 0.0117  (-0.00746 – 0.0309) | 0.0111  (-0.00855 – 0.0308) |
| Moderate-to-vigorous physical activity | 0.0427  (-0.0678 – 0.153) | 0.0459  (-0.0783 – 0.170) |
| **Leisure hours** |  |  |
| Sedentary behaviour | -0.580  (-1.33 – 0.172) | -0.603  (-1.36 – 0.153) |
| Light physical activity | 0.523  (-0.616 - 1.66) | 0.551  (-0.623 - 1.73) |
| Moderate-to-vigorous physical activity | 0.0489*  (-0.00371 – 0.101) | 0.0454*  (-0.00695 - 0.0977) |
| Observations | 277 | 277 |
| Adjusted model; adjusted for sex, age, obesity, smoking history, education, and cardiovascular diseases * p < 0.10, ** p < 0.05 | | |

| **Table S4.**  Odds of having problems in each of the EeuroQol-5 Dimensions ^a^ between physically inactive compared to active participants ^b^ | | | | | |
| --- | --- | --- | --- | --- | --- |
|  | Mobility | Self-care | Usual Activity | Pain or Discomfort | Anxiety or Depression |
|  | OR (SE) | OR (SE) | OR (SE) | OR (SE) | OR (SE) |
| **Unadjusted model** | | | | | |
| Physically active  (n = 133) | reference | reference | reference | reference | reference |
| Physically inactive  (n = 144) | 1.33 | 2.54 * | **2.10** ** | 1.42 | 1.03 |
|  | (0.813 – 2.18) | (0.880 – 7.33) | (1.15 – 3.83) | (0.881 – 2.28) | (0.640 – 1.65) |
| **Adjusted model** | | | | | |
| Physically active  (n = 133) | reference | reference | reference | reference | reference |
| Physically inactive  (n = 144) | 1.11 | 1.46 | 1.89 * | 1.50 | 1.05 |
|  | (0.641 – 1.91) | (0.456 – 4.67) | (0.982 – 3.62) | (0.901 – 2.48) | (0.631 – 1.75) |
| ^a^ Participants were categorised into either ‘Having problem’ (1) or ‘Having no problem’ (0) in each dimension  ^b^ physically inactive refers to participants who did not meet the current physical activity guideline (≥150 minutes moderate-intensity or >75 minutes vigorous-intensity equivalent physical activity per week), while active refers to participants who met the guideline  * p < 0.10, ** p < 0.05 | | | | | |

| **Table S5** Odds of having problems in each of the EeuroQol-5 Dimensions^a^ between sedentary compared to not-sedentary participants ^b^ | | | | | |
| --- | --- | --- | --- | --- | --- |
|  | Mobility | Self-care | Usual Activity | Pain or Discomfort | Anxiety or Depression |
|  | OR (SE) | OR (SE) | OR (SE) | OR (SE) | OR (SE) |
| **Unadjusted model** | | | | | |
| Not-sedentary  (n = 188) | reference | reference | reference | reference | reference |
|  |  |  |  |  |  |
| Sedentary  (n = 89) | 1.45 | 1.76 | 1.22 | 1.67 * | 1.06 |
|  | (0.860 – 2.43) | (0.669 – 4.62) | (0.666 – 2.23) | (0.995 – 2.81) | (0.639 – 1.76) |
| **Adjusted model** | | | | | |
| Not-sedentary  (n = 188) | reference | reference | reference | reference | reference |
|  |  |  |  |  |  |
| Sedentary  (n = 89) | 1.32 | 1.36 | 1.26 | **1.72** ** | 1.02 |
|  | (0.755 – 2.31) | (0.467 – 3.97) | (0.661 – 2.40) | (1.01 – 2.94) | (0.602 – 1.73) |
| ^a^ Participants were categorised into either ‘Having problem’ (1) or ‘Having no problem’ (0) in each dimension  ^b^ Sedentary refers to spending at least nine hours per day in sedentary behaviours, while not-sedentary refers to spending less than nine hours per day in sedentary activities  * p < 0.10, ** p < 0.05 | | | | | |

**Figure S1.** Participants reporting problems in each of the EeuroQol-5 Dimensions ^a^ compared between physically inactive and active participants ^b^

^a^ Health-related quality of life dimensions were collected using EQ-5D-5L interviewer-administered questionnaire. Participants were categorised into either ‘Having problem’ (1) or ‘Having no problem’ (0) in each dimension

^b^ physically inactive refers to participants who did not meet the current physical activity guideline (≥150 minutes moderate-intensity or >75 minutes vigorous-intensity equivalent physical activity per week), while active refers to participants who met the guideline

**Figure S2.** Participants reporting problems in each of the EeuroQol-5 Dimensions ^a^ compared between sedentary and not-sedentary participants ^b^

^a^ Health-related quality of life dimensions were collected using EQ-5D-5L interviewer-administered questionnaire. Participants were categorised into either ‘Having problem’ (1) or ‘Having no problem’ (0) in each dimension

^b^ Sedentary refers to spending at least nine hours per day in sedentary behaviours, while not-sedentary refers to spending less than nine hours per day in sedentary activities
